# Differential Virulence and Pathogenesis of West Nile Viruses

**DOI:** 10.3390/v5112856

**Published:** 2013-11-22

**Authors:** Emilie Donadieu, Céline Bahuon, Steeve Lowenski, Stéphan Zientara, Muriel Coulpier, Sylvie Lecollinet

**Affiliations:** Université Paris Est Créteil (UPEC), UMR 1161 Virologie, Institut National de la Recherche Agronomique (INRA), Agence nationale de sécurité sanitaire de l’alimentation, de l’environnement et du travail (ANSES) , Ecole Nationale Vétérinaire d’Alfort (ENVA), 7 avenue du Général De Gaulle, Maisons-Alfort 94700, France; E-Mails: donadieuemilie@yahoo.fr (E.D.); celine.bahuon@anses.fr (C.B.); steeve.lowenski@anses.fr (S.Lo.); stephan.zientara@anses.fr (S.Z.); mcoulpier@vet-alfort.fr (M.C.)

**Keywords:** West Nile virus strains, central nervous system, inflammation, neuronal injury, immunity, virulence, pathogenesis

## Abstract

West Nile virus (WNV) is a neurotropic flavivirus that cycles between mosquitoes and birds but that can also infect humans, horses, and other vertebrate animals. In most humans, WNV infection remains subclinical. However, 20%–40% of those infected may develop WNV disease, with symptoms ranging from fever to meningoencephalitis. A large variety of WNV strains have been described worldwide. Based on their genetic differences, they have been classified into eight lineages; the pathogenic strains belong to lineages 1 and 2. Ten years ago, Beasley *et al.* (2002) found that dramatic differences exist in the virulence and neuroinvasion properties of lineage 1 and lineage 2 WNV strains. Further insights on how WNV interacts with its hosts have recently been gained; the virus acts either at the periphery or on the central nervous system (CNS), and these observed differences could help explain the differential virulence and neurovirulence of WNV strains. This review aims to summarize the current state of knowledge on factors that trigger WNV dissemination and CNS invasion as well as on the inflammatory response and CNS damage induced by WNV. Moreover, we will discuss how WNV strains differentially interact with the innate immune system and CNS cells, thus influencing WNV pathogenesis.

## 1. Introduction

West Nile virus (WNV) is a member of the Japanese encephalitis (JE) antigenic complex and belongs to the *Flavivirus* genus in the *Flaviridae* family, which includes other major human pathogens such as the Saint Louis encephalitis, Japanese encephalitis, yellow fever, and dengue viruses. Flaviviruses consist of enveloped particles that surround ssRNA+ genomes of about 11 kb. The WNV genome comprises a single open reading frame that codes for three structural proteins, the envelope protein (E), the precursor membrane (prM), and the capsid (C), as well as at least seven non-structural (NS) proteins (NS1, 2A, 2B, 3, 4A, 4B, and 5) [1]. WNV was first isolated from the blood of a febrile woman in Uganda in 1937 [2] and currently has a worldwide distribution that ranges from Africa, the Middle East, Europe, Asia, and Oceania to South and North America. 

WNV is maintained in an enzootic cycle between mosquitoes and birds [3] but can also infect and cause disease in other vertebrate animals, including horses and humans. In most humans, WNV infection is subclinical, but approximately 20%–40% of those infected may develop symptoms of WNV disease ranging from West Nile fever (fever, headache, malaise, lymphadenopathy, myalgia, fatigue, skin rash, diarrhoea, and vomiting) to meningoencephalitis (muscle weakness, tremors, paralysis, and cognitive impairment) or flaccid paralysis (a polio-like syndrome), and, less frequently, death [1,4,5,6]. Hepatitis, pancreatitis, and myocarditis have also infrequently been described to occur [1]. In addition, long-term sequelae, including weakness, persistent movement disorders, and cognitive deficits, frequently occur in patients that have suffered from West Nile neuroinvasive disease [7,8,9,10,11]. Although inactivated and recombinant vaccines are available for animal use, no vaccines or antiviral therapies are currently approved for humans [12]. 

**Figure 1:**
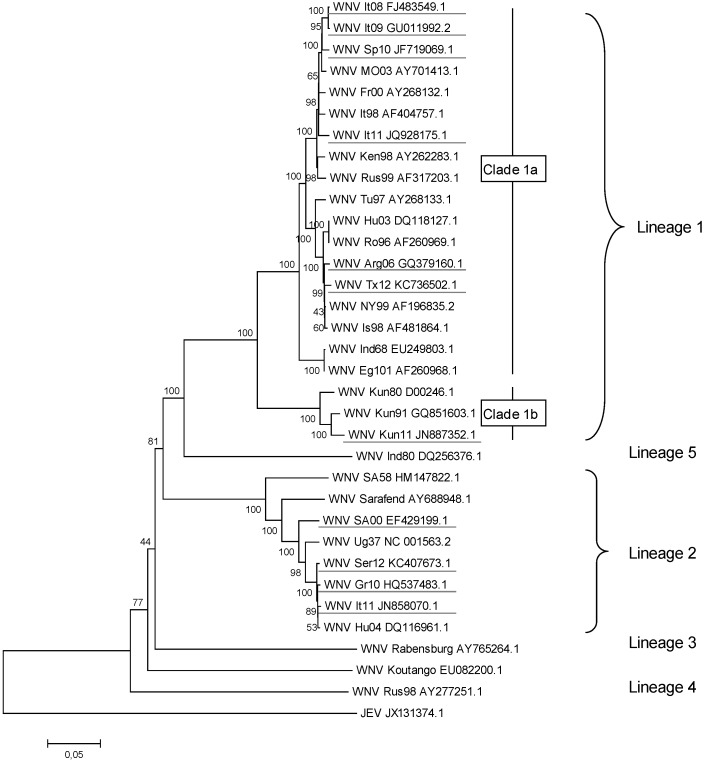
West Nile Virus (WNV) genetic diversity, evaluated using genetic alignment of complete genomic sequences. GenBank accession numbers are indicated on the tree branches of each virus; the first two or three letters stand for the country or the USA state reporting WNV (It = Italy, Sp = Spain, Mo = Morocco, Fr = France, Ken = Kenya, Rus = Russia, Tu = Tunisia, Hu = Hungary, Ro = Romania, Arg = Argentina, Tx = Texas, NY = New York, Is = Israel, Ind = India, Eg = Egypt, Kun = Kunjin Australia, SA = South Africa, Ug=Uganda, Ser = Serbia, and Gr = Greece) and the numbers indicate the year of isolation (96 = 1996, 10 = 2010). Japanese encephalitis virus (JEV), a closely related flavivirus, was used as an outgroup. The rooted phylogenetic tree was constructed using neighbor-joining with Jukes-Cantor parameter distances (scale bar) in MEGA (MEGA software, version 5.2 [25]). A bootstrapped confidence interval (1,000 replicates) and a confidence probability value based on the standard error test were also calculated using MEGA. The WNV strains responsible for recent human or equine outbreaks are underlined. The complete sequences of the most recent Romanian and Russian lineage 2 variants are not available, but at least two introduction events of lineage 2 strains have occurred in Europe: divergent lineage 2 strains have been observed in Romania/Russia and Hungary/Greece/Italy/Serbia/Austria [26].

Over the last 20 years, several outbreaks in humans have been reported in the Mediterranean basin and southern Europe, with fatal cases of encephalitis occurring mainly among elderly people. Outbreaks in humans have occurred in Algeria (1994), Romania (1996–2009), Tunisia (1997, 2012), the Czech Republic (1997), Israel (1999–2000, 2005–2010, 2012), Russia (1999–2001, 2004–2007, 2010–2013), Morocco (1996), France (2003), Hungary (2003–2013), Portugal (2004), Spain (2004, 2010), and Italy (2008–2013) in the 1990s and 2000s [13,14,15,16]. The different strains that caused these epidemics belong mainly to clade 1a and are grouped into the Israeli/American (Is98, Tu97, Hu03, Ro96) or the Kenyan/Western Mediterranean (Mo96, It08, It09, Sp10) clusters (Figure 1). Although lineage 2 strains were initially considered to be of low virulence, they have caused epidemics in eastern and southern Europe since 2008 (Gr10, It11, Ser12) [17]; numerous human cases due to lineage 2 infections were reported in 2010 in Greece (197 human cases, 33 deaths), Romania (57 human cases, 5 deaths), and Russia (480 human cases, 6 deaths) [18,19,20,21]. South Africa and Australia have concurrently and similarly reported an increase in the virulence of lineage 2 and 1b strains (Kun11), respectively [22,23,24], which underscores how the plasticity and adaptive capacity of WNV result in a continuous risk whereby WNV genotypes with enhanced virulence for vertebrates will emerge. 

The most striking example of WNV emergence and pathogenicity can be seen in the introduction and spread of WNV in the New World. A highly virulent WNV strain from clade 1a (NY99) was introduced into New York City during the summer of 1999. The virus then spread rapidly across North, Central, and South America [27], causing severe neurological illness and death in humans and horses and affecting wild bird populations, in particular the American crow (*Corvus brachyrhynchos*), mainly in the USA and Canada [13,27,28]. As of February 2013, 36,801 human cases, including 1,506 deaths (4.1% lethality), had been reported to the Centers for Disease Control and Prevention (CDC, Atlanta, GA, USA). Although a declining trend in the number of human WNV neuroinvasive cases has been observed since 2003, the USA suffered in 2012 from one of the most severe epidemics ever reported, which resulted in 5,674 cases and 286 deaths (compared with 9,862 cases and 264 deaths in 2003). Exceptional weather conditions and, specifically, an unusually warm and dry summer may have accounted for the extreme transmission pattern [29,30]. As a result of the emergence of WNV in the Western Hemisphere, extensive experimental studies with North American WNV isolates (such as NY99, Figure 1) have been conducted, and significant progress in dissecting the viral and host factors that determine the pathogenesis and outcome of WNV infections is being made (for recent and comprehensive reviews, refer to [31,32,33,34,35,36]). However, at present, a more precise understanding of the comparative pathogenesis of the WNV strains that circulate worldwide is needed and would certainly help in explaining observed differences in the outcomes of WNV infections [27].

When WNV infection results in neuroinvasive disease, the virus induces inflammatory lesions and neuronal damage in several brain regions such as the hippocampus, the brainstem, the cerebellum, and the anterior horn of the spinal cord [1,5,37,38]. The mechanisms by which WNV induces neuronal injury are still unclear. Although neuronal injury may be directly caused by viral infection, it may also results from leukocyte infiltration and the host inflammatory response [34,39]. In-depth characterization of WNV pathogenesis provides an opportunity to enhance our understanding of the pathophysiology and immunopathology of acute viral encephalitis. In this review, we will summarize our current knowledge on the factors that trigger WNV dissemination and invasion of the central nervous system (CNS) as well as our understanding of the processes that provoke inflammatory responses and damage in the brains of infected mammals. Moreover, we will discuss how genetically diverse WNV strains can differentially interact with the innate immune system and CNS cells, thus influencing WNV pathogenesis.

## 2. WNV Genetic Diversity

Even though WNV has a single serotype, it nonetheless exhibits considerable genetic variation [40]. Using genotyping, WNV isolates can be classified into at least eight different lineages; lineages 1 and 2, the two major lineages, have been responsible for outbreaks in humans and equines. Lineage 1 comprises most of the isolates responsible for outbreaks in Africa, Europe, the Middle East, Asia, Oceania (Kunjin strains), and North America. Lineage 2 primarily consists of strains isolated in Africa and has historically been considered to be less pathogenic in humans than lineage 1. However, since 2004, bird of prey mortality (and more specifically that of goshawks, *Accipiter gentilis*) has been caused by a lineage 2 strain found in central Europe, in Hungary, which highlights this lineage’s potential to spread outside of Africa and to become virulent in vertebrates [41]. More recently, and as briefly described in the introduction, lineage 2 strains have been responsible for growing numbers of WNV outbreaks in humans and equines in South Africa, as well as in eastern and southern Europe [17,19,20,21,22,23,24,42,43]. 

Other genetic lineages, lineages 3–8 that show 20%–30% genetic divergence from lineages 1 and 2 have been described more recently (Figure 1). Lineage 3 consists of the Rabensburg strain isolated from *Culex pipiens* mosquitoes in central Europe (Czech Republic) [44], and lineage 4 consists of the LEIVKrnd88-190 strain isolated from *Dermacentor marginatus* ticks in Russia [45]. Isolates collected from mosquitoes (mainly *Culex* and *Anopheles* spp.) and humans in the 1950s and 1980s in India form a distinct genetic lineage, lineage 5, while the putative lineages 6, 7, and 8 include, respectively, the Sarawak viruses found in Malaysia, the Koutango viruses found in Senegal, and a new virus found in Spain for which partial sequences have been obtained from *Culex pipiens* [40,46,47,48]. 

Finally, WNV demonstrates broad genetic and geographical diversity within the African continent, which is the source of all the strains found around the globe [26,49]. While single introduction events took place in India, Australia, and the Americas, leading to the subsequent establishment and spread of WNV in these regions and the spatial categorization of WNV strains (clades 1b and lineage 5 in particular) [49], WNV strains were introduced into Europe and the Middle East on several occasions by migratory birds [49,50,51,52,53]. An in-depth understanding of the diversity and evolution of African WNV strains is essential since WNV is increasingly being found in populated areas of North America and Europe.

Moreover, WNV demonstrates significant antigenic diversity, as evidenced in neutralization assays employing monoclonal and polyclonal antibodies [46,54,55]. Specifically, several lineage 1 and lineage 2 isolates differ in the expression or accessibility of epitopes within subdomain 3 of their envelopes [56]. 

Interestingly, the virulence levels of WNV strains do not track their genetic lineages. Beasley *et al.* [57] compared the genetic characteristics and virulence of 19 WNV strains belonging to lineages 1 and 2. Within each of those lineages, WNV strains demonstrated significant differences in virulence and neuroinvasiveness in experimentally infected mice and hamsters. In particular, two Kunjin isolates (clade 1b) and three lineage 2 strains from Cyprus and Madagascar were found to be non-neuroinvasive or highly attenuated in mice, while other lineage 1 and lineage 2 strains were highly neuroinvasive. The differential virulence of lineage 1 strains in WNV amplifier birds, e.g., American crows (*Corvus brachyrhynchos*) and house sparrows (*Passer domesticus*), was also manifest; a WNV strain from Australia (KUN6453) caused diminished and delayed morbidity and mortality compared to that caused by isolates from North America (NY99) [58,59]. Ken98 and NY99 strains had comparable virulence in mice and house sparrows, while Ken98 was clearly attenuated in American crows, which suggests that specific WNV strains may have differential pathogenesis in different bird and mammal hosts.

Several studies have sought to decipher the factors that mediate the differences in virulence among WNV strains. Because most Kunjin isolates, in contrast to the highly virulent lineage 1a strains, do not have glycosylation sites (a N-Y-S tripeptide located at positions 154–156 in the envelope protein), it was hypothesized that the glycosylation status of the envelope protein may play an important role in determining WNV virulence [60,61,62,63,64,65]. Several studies have suggested that envelope glycosylation significantly contributes to the enhanced virulence of the North American WNV strains in mammals and birds [63,64,65] and to the efficacy of WNV transmission by mosquito vectors [66]. Other virus determinants may have a critical influence on the virulence of lineage 1 strains; for instance, the presence of a proline residue at position 249 in the NS3 gene (present in the highly virulent NY99 strain and replaced by a threonine in the less virulent Ken98 strain) greatly enhances WNV replication and virulence in the American crow (*Corvus brachyrhynchos*) [67]. The presence of an isoleucine residue at position 141 in the prM [65] as well as an alanine residue at position 30 in the NS2A gene [68,69] have also been shown to enhance virulence for American crows and house sparrows. Botha *et al.* (2008) demonstrated that highly virulent and less virulent lineage 2 strains differed in their 3' non-coding regions (deletions had occurred in the less virulent strains) and non-structural genes (specifically, NS5 was the most variable) [70]. Reverse genetics has been used to perform controlled exchanges of genomic fragments between lineage 1 (NY99, Kunjin) and lineage 2 (W956) strains, and the results underscore the role WNV non-structural proteins play in WNV virulence [68,71]. 

Elucidating WNV pathogenesis in humans has proved to be difficult because WNV strains demonstrate variable virulence in mammals; moreover, asymptomatic or subclinical infections are highly prevalent and confirmed human infections are infrequent, particularly outside the USA. Animal models of WNV infection, including mice, hamsters, and non-human primates [72], have provided insights into the pathogenesis of WNV in mammals. In particular, infecting wild type or genetically engineered mice with WNV has enabled the scientific community to identify viral and host factors that control WNV dissemination and entry into the CNS [34,72,73].

## 3. WNV Dissemination and Entry into the Central Nervous System

**Figure 2:**
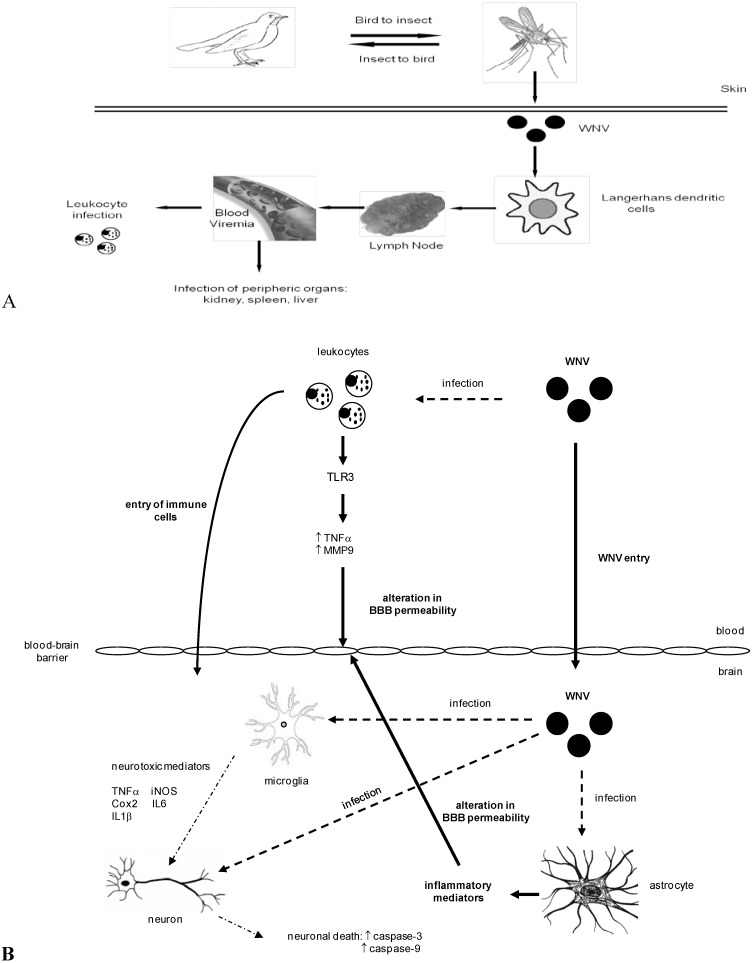
(**A**) Simplified schematic of the initial pathogenesis of WNV infections after inoculation of the virus into the skin. After intradermal inoculation by infected mosquitoes, WNV replicates in skin cells, specifically in Langherhans cells (LCs). Infected LCs migrate to regional lymph nodes and pass through efferent lymphatic vessels to reach the bloodstream. This primary viremia results in the infection of leukocytes and of peripheral tissues such as the spleen, liver and kidneys; (**B**) Simplified schematic of the regulation of BBB permeability following WNV infection and entry into the brain. WNV infects and replicates in leukocytes, which act as reservoirs for the virus in a Trojan Horse-like fashion. Viral sensors (such as TLR3) recognize WNV and initiate the production of matrix metalloproteinases, such as MMP9, and pro-inflammatory cytokines, such as TNFα, which have both been shown to increase BBB permeability and facilitate WNV dissemination into the brain. Infected microglia respond by releasing pro-inflammatory mediators (TNFα, iNOS, Cox2, IL6, and IL1β), which cause neuronal death. Infected astrocytes also produce inflammatory mediators that can amplify the local immune response and modify BBB permeability. Neuronal death results, in large part, from an upregulation of the apoptotic caspase-3 and caspase-9 pathways following infection with North American isolates. BBB: blood-brain barrier, TLR3: Toll-like Receptor 3, MMP9: matrix metalloproteinase 9, TNFα: tumor necrosis factor alpha, iNOS: nitric oxide synthase, Co-2: cyclooxygenase 2, IL6: interleukin 6, IL1β: interleukin 1 beta.

After an organism is intradermally inoculated with WNV by infected mosquitoes, WNV is thought to initially replicate in keratinocytes, newly recruited neutrophils and skin dendritic cells, specifically in Langherhans cells (LCs) [74,75,76]. Infected LCs rapidly become fully functional antigen-presenting cells as major histocompatibility class II (MHC) and co-stimulatory molecules such as CD54 (ICAM-1, Intercellular Adhesion Molecule-1) and CD80 (B7-1) are expressed [77]. Infected LCs and neutrophils migrate to regional lymph nodes and pass through efferent lymphatic vessels to reach the bloodstream. This primary viremia (3–4 days post-infection in mammals) contributes to early virus spread and results in the infection of peripheral tissues such as the spleen, liver, and kidneys [34,74] (Figure 2A). In the draining lymph nodes, antiviral cytokine and chemokine expression, complement activation, and viral antigen presentation to naive T-lymphocytes are induced [78]. By the end of the first week after WNV inoculation, WNV is cleared from the blood and peripheral organs, and infection of the CNS can be observed starting at day 5 post-infection, provided that sufficient peripheral replication of WNV has occurred [34,79,80,81]. 

In peripheral tissues, virus infection and dissemination are limited by early innate immune responses and especially by the antiviral interferon (IFN) type I response [31,35]. Ample experimental evidence shows that the type I IFN response is a key innate defence mechanism. WNV replication can be inhibited *in vitro* by pre-treating cells with type I IFNs [82,83]. *In vivo*, rodents can be protected against lethal WNV infection if they are treated with type I IFNs, and mice genetically deficient in type I IFN signalization pathways are more sensitive to WNV infection [82,84]. Remarkably, resistance to type I IFNs has been shown to affect WNV replication fitness and virulence [85]. Several WNV non-structural proteins, namely NS2A, NS4B, and NS5, are known to counteract the type I IFN response through diverse effector mechanisms. NS2A inhibits IFN-β transcription and expression [86], while NS4B and NS5 block IFN signaling pathways by inhibiting STAT1 and STAT2 or JAK1 and TYK2 phosphorylation, respectively, downstream of type I IFN expression [87,88,89]. The attenuated phenotype shown by the lineage 1 Kunjin strains or the MAD78 strain from Madagascar can at least partially be explained by the strains’ higher degree of sensitivity to type I IFNs [90]. These strains have been shown to be defective in their ability to suppress the IFN-signalling Jak-Stat pathway, including nuclear translocation and the accumulation of STAT proteins [85,90]. A point mutation in the gene coding for NS5 (NS5_S653F_) in the Kunjin strains accounts for this high degree of sensitivity to type I IFNs [89].

WNV dissemination is also countered by the effector functions of innate and adaptive immune cells (γ/δ T cells, NK cells, neutrophils, macrophages, and IgM-secreting B cells) [31,74,91,92,93,94,95]. While NK cells do not seem to play a significant role in either WNV control or pathogenesis in mice, the γ/δ T cell population has been shown to grow after WNV infection and to be indispensable in controlling WNV replication and dissemination because γ/δ T cells produce high levels of IFN-γ [93,94,96]. In addition, γ/δ T cells help shape adaptive immune responses against WNV by facilitating the maturation of professional antigen-presenting cells, e.g*.*, DCs [97]. Macrophages are effective antigen-presenting cells; they promote the proliferation of WNV-specific T cells [98] and the production of reactive oxygen intermediates, such as nitric oxide, which can also directly inhibit WNV replication [99,100]. The protection they provide against WNV infection has been demonstrated *in vivo* in mice [101]. 

Of paramount importance to the clinical outcome of a case is whether or not WNV gains entry to the CNS; nonetheless, it is important to note that WNV antigens and inflammatory infiltrates can be present and neuronal damage can occur in the CNS over the course of subclinical infections [72,102]. Indeed, the ability of WNV strains to enter the CNS is thought to determine their virulence and results in their classification as highly, mildly, or non-neuroinvasive [57]. The mechanisms by which WNV enters the CNS are not fully understood and may depend on the infection route and the pathogenicity of the WNV strain [57]. Three major routes of CNS entry have been proposed: (i) entry through the blood-brain barrier (BBB) via infected leukocytes; (ii) direct crossing of the BBB after its integrity has been compromised or the brain endothelial cells have become infected; or (iii) entry by retrograde axonal transport after the peripheral nervous system has become infected [31,81,103,104]. 

Bidirectional axonal transport of WNV has been demonstrated in hamsters [103]. Retrograde axonal transport allows virus entry into the CNS and accounts for acute limb paralysis, while anterograde transport would facilitate WNV spread in the CNS.

However, the first two hypotheses, involving WNV entry through the BBB, are the best-supported ones. The BBB is a highly regulated interface between the blood and the brain and is composed of a tight endothelium, a basement membrane (composed of collagen IV, laminin, proteoglycans, and glycoproteins), pericytes, and astroglial feet [105]. The BBB restricts access of pathogens, immune cells, and immune mediators to the brain, thus preventing infection and limiting the potential side effects of immune system activation on generally non-renewable neurons [106,107]. However, WNV has been found to induce the expression of cell adhesion molecules, such as ICAM-1, VCAM-1 (Vascular Cell Adhesion Molecule-1), and E-selectin, at the surface of the endothelium, thereby facilitating the entry of immune cells and, consequently, the virus, in a Trojan Horse-like fashion [104,108]. During peripheral infection, the expression of several cytokines, such as IFN-α, then results in the upregulation of ICAM-1 expression on the luminal surface of the BBB endothelium. This increased expression of ICAM-1 is detectable before WNV enters the brain and plays an important role in virus neuroinvasion *in vivo* (Figure 2B) [109]. Dai *et al.* (2008) showed that ICAM-1 knock-out (KO) mice had increased survival rates that were associated with significantly lower virus burdens and significantly fewer brain lesions, as well as decreased BBB leakage, following a lethal WNV challenge [110]. These latter results, put in the context of other studies [111,112,113], suggest that ICAM-1 acts both as a ligand for leukocyte receptors at the surface of the BBB endothelium and as a signal transducer that influences BBB permeability and the neuroinflammation process, thus facilitating the transmigration of infected leukocytes. 

Nonetheless, the relationship between BBB disruption and WNV-induced encephalitis is strongly debated at present [114,115,116], and free WNV particles can also access and cross the BBB without compromising its integrity [104]. It has been frequently documented that WNV infection alters the expression of several proteins involved in either the maintenance of the BBB’s integrity or the trafficking of leukocytes. MMP9 (matrix metalloproteinase 9), a factor that is secreted by activated innate immune cells, plays an important role in WNV encephalitis as both its expression and its enzymatic activity are rapidly upregulated following WNV infection [33,117]. Furthermore, the resistance of MMP9 KO mice to WNV disease seems to stem from the uncompromised integrity of their BBBs; they have significantly lower brain viral loads and lower levels of inflammatory infiltrates [117]. It can be hypothesized that activated matrix metalloproteinases degrade the extracellular matrix of the BBB, thereby facilitating the entry of WNV particles. Inflammatory cytokines are also important modulators of BBB permeability [118]. Among them, the tumor necrosis factor alpha (TNFα) protein and the macrophage inhibitory factor (MIF) protein are the most studied [116,119,120]. In experimentally infected mice and naturally infected humans, plasma levels of MIF were found to increase after WNV infection. Moreover, neuroinvasion was delayed and reduced in MIF KO mice compared to wild-type mice [119]. Studies investigating the role of Toll-like receptor 3 (TLR3), a dsRNA sensor expressed on the surface of macrophages, neurons and microglia [94], have found that TNFα is key in modulating BBB permeability and altering the tight junctions between endothelial cells. TLR3 senses the dsRNA molecules produced during WNV replication and subsequently mediates the secretion of pro-inflammatory cytokines, including interleukin 6 (IL6) and TNFα. Wilson *et al*. (2008) tested the individual capacity of WNV non-structural proteins to inhibit the signal transduction mediated by TLR3; they observed that NS1 inhibits the activation of the TLR3 pathway [121]. It is interesting to note that conflicting results have been obtained in TLR3 KO mice; either virus replication in the brain is enhanced and BBB permeability is not altered [115] or BBB integrity is better maintained and the survival rate is enhanced [116]. These contrasting outcomes could result from differences in the extent and duration of cytokine secretion following TLR3 engagement. In an attempt to explain why WNV neuroinvasive forms mainly occur in older patients, the regulation of TLR3 expression on the surface of primary human macrophages was compared for macrophages from younger and older blood donors following WNV infection. This study revealed that the cells of older donors had elevated TLR3 levels and elevated cytokine levels, which may contribute to an increase in BBB permeability and thus disease severity in older individuals [122]. 

Finally, peripheral pro-inflammatory cytokines are major modulators of BBB permeability, and, interestingly, mice infected with seven strains from lineages 1 and 2 that had varying virulence had different types and levels of pro-inflammatory cytokines (mainly IFNs and accordingly IFNs induced proteins or IL6) in their livers and spleens [123]. This study suggests that different WNV strains may interact differently with the host’s peripheral innate immune system. 

## 4. WNV Neuropathogenesis

Once WNV has entered the brain, it causes encephalitis by virtue of its ability to infect and injure neurons through direct (induced by the virus) and indirect (or bystander, induced by the immune response) mechanisms [124]. 

WNV primarily targets neurons found in the diverse structures of the brain, notably the cortex, brainstem, and hippocampus (especially pyramidal neurons), leading to their alteration or death [37,39,125]. Infection and apoptosis of motor neurons in the anterior horn of the spinal cord result in flaccid paralysis in humans and rodents [1,103,126], and variable anatomical involvements could explain the different clinical presentations of WNV. For instance, WNV tropism for specific brain areas may vary depending on the inoculation route (intracerebral *vs*. footpad inoculation), dose, or strain [127,128,129]; in contrast, the type of cell line (mammalian *vs*. insect) used for *in vitro* viral growth does not significantly affect WNV pathogenesis [130].

**Figure 3:**
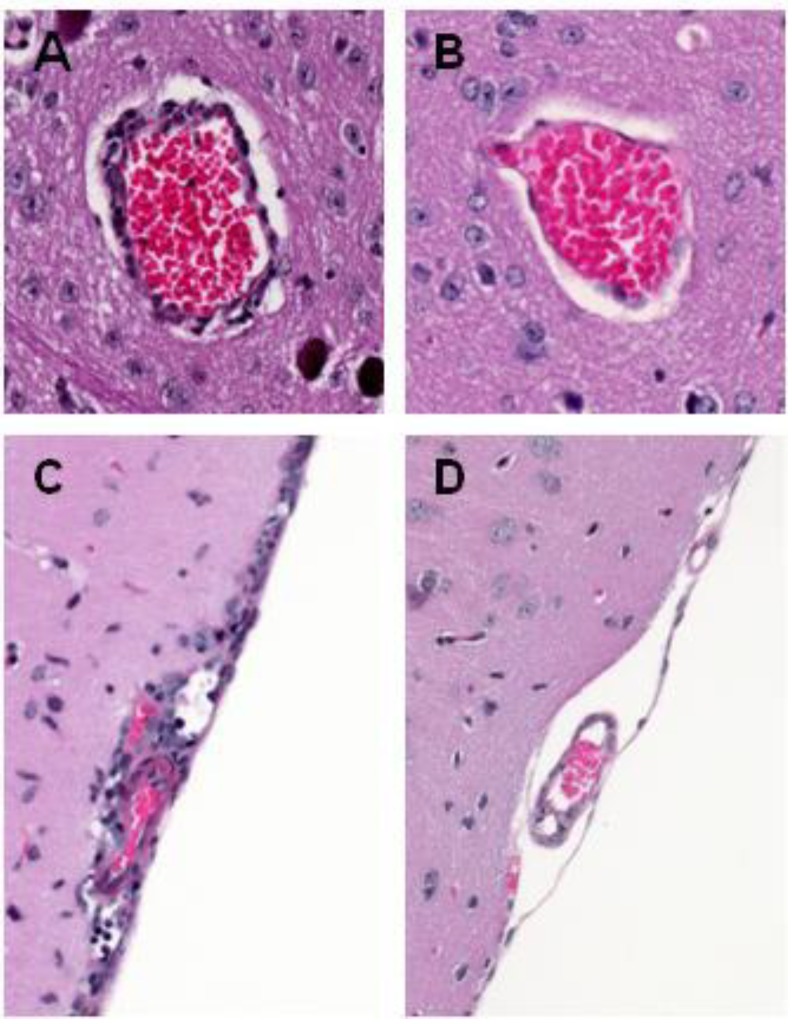
Representative histologic lesions in the brains of mice infected with WNV. (**A**) Haematoxylin- and eosin-stained section showing perivascular infiltrates of lymphocytesand macrophages (WNV infection) (×20 magnification); (**B**) Haematoxylin- and eosin-stained section showing a control blood vessel (without perivascular infiltrates, Control) (×20 magnification); (**C**) Haematoxylin- and eosin-stained section showing mild meningitis (WNV infection) (×20 magnification). (**D**) Haematoxylin- and eosin-stained section showing control meninges (without infiltrate, Control) (×20 magnification).

Microscopic examination of human and rodent brains reveals that histological changes occur that are consistent with the manifestation of clinical disease [131]. These changes include the activation of resident microglia; perivascular and parenchymal accumulation of macrophages, CD4^+^ and CD8^+^ T cells, and B cells; formation of microglial nodules; reactive proliferation of astrocytes; and eventual neuronal cell death (Figure 3) [132,133,134,135]. The lesions that result from neuron death, which can be patchy in distribution, occur in the brainstem, cerebral cortex, hippocampus, thalamus, and cerebellum. These observations raise the question as to whether all WNV strains induce histopathological damage and inflammatory lesions of the same type, at the same location, and with the same severity. A study by Donadieu *et al.* found that lineage 1 Mediterranean (Is98) and Australian (Kun35911) isolates, introduced via intraperitoneal inoculation, differed in their neuropathogenesis and, in particular, in the severity and location of the apoptotic and inflammatory lesions [127]. 

WNV induces neuronal damage and loss in several brain regions [5,37,38]. WNV infection has been shown to trigger apoptosis, and *in vivo* and *in vitro* studies have begun to elucidate the molecular pathways involved in WNV-induced cell death. WNV seems to be responsible for caspase-3- and bax-dependent programmed cell death [39,136]. Samuel *et al.* (2007) demonstrated that caspase-3-driven apoptosis could induce WNV neuronal injury and death. Indeed, caspase-3 KO mice had increased survival rates, which were associated with decreased levels of neuronal death in their cerebral cortexes, brainstems, and cerebellums following WNV NY99 infection [39]. However, knocking out caspase 3 did not protect all neuronal populations; in particular, alterations of hippocampal neurons appeared to be largely independent of caspase 3. These results suggest that the molecular pathways involved in WNV-induced neuronal damage could depend on the brain structure or the neurons under consideration. Different WNV strains may also induce apoptotic death using distinct pathways. Indeed, a Kunjin isolate mainly induced caspase-3-independent apoptosis in every brain area investigated, whereas the Is98 strain, genetically close to NY99, induced caspase-3-dependent apoptosis in the cortex and striatum [127]. To evaluate the factors underlying WNV-driven neuropathogenesis, Medigeshi *et al*. (2007) used human neuroblastomas and primary rat neurons derived from the hippocampus [137]. In these *in vitro* models, the presence of WNV non-structural proteins was found to be sufficient to induce pro-apoptotic responses. NS3 in particular induces apoptosis by activating caspases 8 and 3 [138]. The capsid C protein has also been shown to induce rapid nuclear condensation and cell death in a human neuroblastoma cell line, SH-SY5Y [139]. Although WNV-induced neuronal apoptosis has been widely observed, its precise mechanisms are still being explored. Moreover, cell death resulting from WNV infection may be dependent on the infectious dose in that infection with high WNV loads could be more likely to result in necrotic cell death [140,141]. 

**Figure 4:**
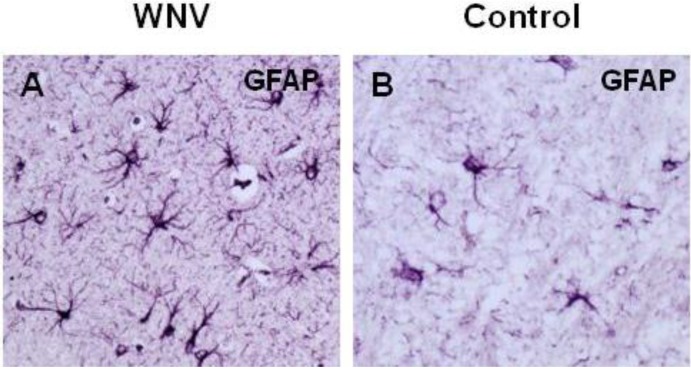
Neuroinflammation in mice infected with WNV. (**A**) A high level of astrocyte reactivity was observed in WNV-infected mice using paraffin-embedded tissue stained for the astrocyte GFAP marker. Astrocytes displayed modified morphologies and hypertrophic cell bodies (×20 magnification); (**B**) A low level of astrocyte reactivity was observed in non-infected mice.

In addition to experiencing direct damage caused by WNV infection, neurons may also undergo apoptosis as a result of bystander effects caused by cytotoxic factors released by neuronal and non-neuronal cells. Pro-inflammatory molecules, such as IL1β, IL6, IL8, and TNFα, may be released by dying neurons and damage non-infected neurons [142]. Non-neuronal cells in the brain, otherwise known as glial cells (e.g*.*, resident microglia and astrocytes), are essential in brain homeostasis and neuronal metabolism and functions [143]; they may also promote WNV infection [132,144]. During WNV-provoked encephalitis, these cells become activated and release excitotoxic amino acids (glutamic and aspartic acids), reactive oxygen species, and pro-inflammatory cytokines that may contribute to WNV pathogenesis (Figure 2 and Figure 4) [145,146,147]. Following flavivirus infection, activated glial cells release TNFα, IL1β, IL6, and RANTES, all of which promote bystander damage to neurons [148,149]. However, while the extent to which glial infection contributes to JEV-induced neurological disease has been well studied, its relevance in WNV-induced disease has received less attention. The role of bystander effects and excessive brain inflammation in brain tissue damage and WNV pathogenesis needs to be thoroughly addressed [150]. In particular, Hussmann *et al.* (2013) suggested that the attenuated phenotype of the lineage 1 MAD78 WNV strain was not due to poor neuroinvasive properties; rather, it likely resulted from the strain’s altered neuropathogenesis and, more specifically, its inability to replicate in non-neuronal target cells within the CNS. Specifically, the study found that astrocytes were less susceptible to MAD78 than to NY99: virus replication was delayed and cell-to-cell spread was reduced [151].

In *in vivo* studies of WNV in rodent models, no clear correlation has been observed between the severity of CNS histological lesions and the intensity of clinical signs [127,152]. Recent investigations of the cause of death in WNV-infected mice and hamsters suggest that the rodents died from respiratory insufficiency, which preceded death by up to four days [153]. Focalized neuropathy in respiratory control centers in the medulla may have contributed to the animals’ respiratory insufficiency; this finding sheds new light on WNV pathogenesis in rodents. Complementary studies in WNV-infected patients are crucial in revealing if such a mechanism also applies in humans. 

Interestingly, WNV has been shown to persist in experimentally infected monkeys, hamsters, and mice [31,72,102,152,154]; in humans, persistence has only been reported in peripheral organs (e.g., kidneys) [155] and remains controversial. Infectious WNV has been recovered for at least 5.5 months from the cerebellum and subcortical ganglia of rhesus macaques, for 86 days from the brains of golden hamsters, and for up to four months from C57Bl6/J mice. It is worth noting that persistent viral infection and neuronal damage occur at similar levels in mice and hamsters that succumb to WNV infection and in those that survive [73,102,154]. Strikingly, WNV Kunjin isolates display unique persistence properties in mice that are naturally resistant to flaviviruses, including those that demonstrate resistance to the highly virulent lineage 1 WNV strains [152]. Such persistence results from the fact that WNV is not completely cleared from the brain at late time points, demonstrating once again that WNV Kunjin and NY99 strains interact differently with CNS cells. Moreover, the persistence of Kunjin strains contributes to late-occurring pathogenesis and is associated with fatal outcomes.

## 5. WNV Clearance from the Brain

Since neurons regenerate poorly or not at all, controlled and fine-tuned immune responses aim to limit the spread of and eliminate WNV while minimizing neuronal damage. Both innate and cellular immune responses in the CNS orchestrate WNV clearance in animal models [31,34,94,156]. Type I IFNs were shown to be essential in clearing WNV from the CNS as well as in controlling the spread of WNV at the periphery. Abundant literature on mice in which the expression of type I IFNs, their receptors (IFNAR), or downstream signaling of the IFN-IFNAR interaction is knocked out shows that viral replication is enhanced in the CNS and mortality rates increased after WNV infection in KOs [82,157,158]. In contrast, control of WNV infection in the CNS does not appear to involve IFNγ. Following intracranial infection, IFNγ KO mice did not demonstrate diminished levels of viral clearance from their CNSs nor did they experience enhanced lethality [95]. 

Several studies suggest that T-cell-mediated immunity is indispensable in the control of WNV infection in the CNS. Following WNV infection, T-cells and macrophages travel into the brain and participate in viral clearance [96,129,159,160,161]. In CD8^+^ T-cells KO mice as well as in mice that failed to present MHC class I antigens or to demonstrate CD8^+^ T-cells effector mechanisms (perforin KO mice), detectable WNV persisted in the brain for up to 30 days post infection [96,159]. Following WNV infection in mice, CD8^+^ T cells predominate over CD4^+^ T cells in traffic to the brain [129,133,159]. In response to WNV infection, neurons secrete CXCL10 chemokines, which in turn recruit effector CD8^+^ T cells through interactions with their ligands, CXCR3 chemokine receptors. Mice deficient in CXCL10 demonstrated enhanced WNV-induced mortality, increased viral loads, and reduced T-cell trafficking in their brains [162]. The same results were seen in mice without CCR5 chemokine receptors; these receptors also promote T-cell traffic into the brain following WNV infection [160]. Similarly, the presence of the CCR5 loss-of-function allele (CCR5Δ32) in humans is associated with an increased risk of WNV neuroinvasive disease [163]. In contrast, T-cells are sequestered in perivascular spaces as a result of interactions between T-cell CXCR4 and endothelial CXCL12, which in turn restricts virus clearance [79]. Just as mice with impaired CD8+ T cell recruitment are at greater risk for neuroinvasive disease, humans with impaired T-cell immunity are more likely to incur WNV infections in their CNSs [31]. CD4^+^ T cells orchestrate the adaptive immune response, including both CD8^+^ T-cell cytolysis and antibody synthesis, and display direct antiviral activity [164]. When CD4^+^ T cells are absent, late WNV-specific IgM and IgG secretion and CD8^+^ T cell activity in the CNS are impacted; consequently, mice with a genetic or acquired deficiency in CD4^+^ T cells are severely impaired in their ability to clear WNV from their CNSs [165]. 

Although they play a crucial role in virus clearance, T cells can, conversely, cause irrevocable damage to the host and contribute to WNV disease [129]. Regulatory T cells (Tregs) strongly control cellular immune responses and repress excessive and harmful CD8+ T cell responses. It is interesting to note that Treg levels predict WNV infection outcomes in humans and mice [166]: high Treg levels are associated with asymptomatic WNV infections. The inoculation dose, inoculation route, and WNV strain can influence whether immunopathology in the CNS will occur. Mice lacking CD8^+^ T cells that were infected with attenuated WNV strains—and specifically the lineage 2 Sarafend strain experienced fewer damaging CD8^+^ T cell responses, resulting in decreased morbidity and mortality [129]. 

## 6. Future Perspectives

The use of animal models has fostered a significant understanding of WNV pathogenesis, even if several important questions regarding virus-host interactions remain unanswered. The exact mechanisms that the virus uses to cross the BBB, spread within the CNS, and cause neuronal dysfunction and death require further investigation in both animal models and humans. Given that WNV strains exhibit considerable genetic variation but that a large number of previous studies were conducted with North American isolates, and with the NY99 strain in particular, we would predict that diverse WNV strains should demonstrate differential pathogenesis at the periphery, as already evidenced, and in the CNS (preferential tropism for specific neuron subpopulations, differential induction of apoptotic, necrotic, or inflammatory molecular pathways, *etc*.). Gaining further insight into the diversity of WNV strains should help promote the development of novel therapeutic targets that are adapted to locally circulating strains.
